# Global Hand Surgery Fellowship Education: Program-Reported Characteristics and Competencies

**DOI:** 10.1177/15589447251406922

**Published:** 2026-01-02

**Authors:** Andrew D’Elia, Armaan Dhanoa, Barbara Jemec

**Affiliations:** 1 Temerty Faculty of Medicine, University of Toronto, ON, Canada; 2 University Health Network, Toronto, ON, Canada

**Keywords:** hand surgery, education, fellowship, standardization, website, global

## Abstract

**Background:**

Hand Surgery Fellowship is the mainstay for training of hand surgeons globally. Despite this, a standardized curriculum for hand surgeons has yet to be established, and discrepancies between Hand Fellowship offerings, including operative exposure and program requirements, persist. The purpose of this study was to characterize what Fellowship programs globally promise to teach their Fellows, and how they do this.

**Methods:**

An Internet search for Hand Surgery Fellowship programs was conducted between March 3, 2024 and February 8, 2025. In addition to the primary search, programs were found through the International Federation of Societies for Surgery of the Hand and affiliate society websites. Only Fellowships that were recognized by national regulatory bodies were included.

**Results:**

A total of 218 program websites were identified across the United States, the United Kingdom, Canada, Europe, Australia/New Zealand, Asia, Africa/Middle East, and South America. Mentorship/apprenticeships and rotation-based programs were the most common training approaches. Trauma surgery was a universal focus of Hand Fellowships globally. Programs supervised by orthopedic faculty were more likely to offer training in shoulder, elbow, and arthroscopic surgery, whereas programs supervised by plastics/mixed departments were more likely to include peripheral nerve, congenital, microsurgery, and replantation surgery.

**Conclusions:**

Program websites in various regions globally possess different levels of detail and emphasize different aspects of surgical training in their descriptions. The United States had the most programs and included the greatest level of detail in their program descriptions. Reported obtainable clinical competencies varied with geographical region and faculty specialization. Recommendations are suggested.

## Introduction

Medical education is core to the development of competent and skillful physicians. In subspecialty fields such as surgery of the upper extremity, Fellowship training is often required to obtain further knowledge and skills. Hundreds of Fellowships in hand surgery are available to senior trainees in plastic, orthopedic, and general surgery globally. Training differs, however, especially between institutions and parent surgical disciplines. Variations in educational format, caseloads, hospital attributes, patient population, and other factors result in varying levels of clinical competence among Fellowship-trained hand surgeons.^1^

Efforts by the American Society for Surgery of the Hand (ASSH) to improve the unstandardized landscape of hand surgery training dates back to the 1960s.^2,3^ A number of studies have investigated various aspects of hand surgery training with the aim of optimizing outcomes, including fellowship selection criteria,^4,5^ gender diversity,^6^ and previous residency training.^7,8^ Surveys and case log studies highlighted significant differences in caseloads, procedural exposures, and clinical practice patterns of plastics-trained and orthopedic-trained hand surgeons in the United States.^7-10^ Patel et al^11^ also examined the impact of resident co-learners on fellows’ learning experience. Beyond these studies, minimal work has been done to characterize the features, curricular components, clinical experiences, and level of proficiency offered by Hand Fellowships—particularly outside of the United States.

In 2023, the United States Accreditation Council for Graduate Medical Education (ACGME) published new “common program requirements” for education in hand surgery.^12^ Despite intentions to cultivate what is believed to be the ideal hand surgeon, a formal curriculum, which outlines the requisite skills and capabilities of any high-quality hand surgeon, as well as quality assurance measures, has yet to be adopted on a global scale. Silvestre et al^8^ highlight that the ACGME has yet to implement a minimum number of relevant cases for Hand Fellows. In their discussion of fellowship case minimums, Hines et al^3^ suggest that core hand surgery training competencies are a moving target, with growing emphasis on technical proficiency as opposed to sheer case volume. Ng et al^13^ discuss the growing need for interdisciplinary hand surgery training from both plastic and orthopedic specialties in the United Kingdom.

Previous analyses of the American Hand Fellowship programs and their websites, as well as the ASSH Fellowship directory highlight the importance of program website accessibility and clarity, as today’s residents rely on online content to explore program options and navigate the fellowship application process.^14-16^ Herein we conduct a global review of Hand Surgery Fellowship program websites, including general characteristics, educational format, and objective clinical competencies. The objective of this study is to characterize what is being offered as Hand Surgery Fellowship training as described by program websites globally. The results of this study discuss best practices for development of accreditation criteria and standardized hand surgery training curricula.

## Materials and Methods

### Data Collection

Hand Surgery Fellowship program descriptions were found through the International Federation of Societies for Surgery of the Hand (IFSSH) and its affiliate society websites. In addition, a simple web search using the Google Search engine was conducted from a local address in Toronto, Ontario, Canada using Google Chrome. The free-search strategy comprised the terms “hand surgery” AND (“fellowship” OR “training program”) AND “[names of various countries and continents].” The search was conducted between March 3, 2024 and February 8, 2025. Only Fellowships recognized by national regulatory bodies (eg, hand societies, orthopedics associations) were included. Exclusion criteria included any program website that was not written in English or easily translatable via browser-enabled translation service, or scholarship-based fellowships for research or travel. Programs with broken website links or without any form of description were also excluded. Following data collection, programs found in more than one online location were deduplicated. Programs were grouped into geographical regions and compared on a global scale.

Data on individual program requirements, structure, curriculum content, clinical responsibilities, and objective clinical competencies were collected. Clinical competencies were recorded if the program description detailed the skill as a learning goal or a key exposure of the program—or described a rotation/clinic highlighting the specific skill. In general, data were recorded as available in the program description and/or website. Data points were left blank and omitted from analysis if unavailable, unless otherwise specified in this article. The results of the data were subsequently processed to identify patterns in Hand Fellowship program offerings.

## Results

### General Program Characteristics

**Figure 1.**
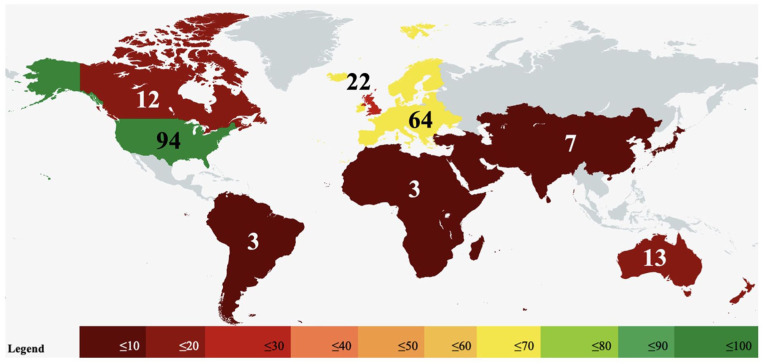
World heat map of Hand Surgery Fellowships. Values represent the number of programs meeting criteria in each of the 8 regions we identified.

The IFSSH and Federation of European Societies for Surgery of the Hand (FESSH) reported 62 and 68 affiliate societies, respectively (Supplementary Table S1). In total, 280 Fellowship programs were identified via affiliate society websites and Google Search. A total of 218 program descriptions meeting our criteria were identified across 8 regions: the United States (94), Europe (64), the United Kingdom (22), Australia/New Zealand (A-NZ) (13), Canada (12), Asia (7), Africa/Middle East (AME) (3), and South America (SA) (3) (Figure 1). Sixty-three programs did not meet our inclusion criteria and were excluded from analysis (Supplementary Table S2). Many more unaccredited fellowships were found globally, but the most common reasons for exclusion were a lack of affiliation with a national regulatory body (59%), travel or research fellowship (16%), or insufficient data (10%).

Tables 1 and 2 describe general characteristics of identified programs. Application processes commonly included presentation of a curriculum vitae, statement or letter of intent, 2 to 3 references, and proof of completion of residency training. Reported funding methods for Fellows included salary equivalent to year of residency training (28), preset stipends (13), self-funded (6) or rarely, independent billing (2). Thirty-two funded programs included benefits such as health and/or malpractice insurance. Some programs included funding for leave (12), conferences (11), and loupes (5).

**Table 1.** Reported Availability and Basic Characteristics of Hand Surgery Fellowships.

Characteristics	A-NZ (n = 13)	Canada (n = 12)	Europe (n = 64)	UK (n = 22)	USA (n = 94)	Asia (n = 7)	SA (n = 3)	AME (n = 3)
% Programs reporting university affiliation	8	100	30	27	55	14	0	33
No. programs reporting position, %	3 (23)	9 (75)	2 (3)	1 (5)	94 (100)	1 (14)	3 (100)	1 (33)
Total no. of reported positions	8	21	12	2	208	4	5	1
Mean no. of reported positions per program (SD)	2.7 (1.15)	2.3 (1.58)	6.0 (4.24)	2.0 (-)	2.2 (1.37)	4.0 (-)	1.7 (0.58)	1.0 (-)
No. Programs reporting duration, %	13 (100)	12 (100)	18 (28)	12 (55)	85 (90)	5 (71)	2 (67)	2 (67)
1-y program, %	7 (54)	9 (75)	3 (5)	5 (23)	85 (90)	1 (14)	0	1 (33)
6-mo program, %	2 (15)	0	2 (3)	0	0	0	0	1 (33)
Variable length program,^a^ %	4 (31)	3 (25)	7 (11)	7 (32)	0	3 (43)	1 (33)	0
Unreported length of program, %	0	0	52 (81)	10 (45)	9 (10)	3 (43)	2 (67)	1 (33)
% Accepting only ortho residents	8	25	0	9	7	0	33	33
% Accepting only plastics residents	0	8	5	5	1	0	0	0
% Accepting only general-trained residents	0	0	0	0	0	0	0	0
% Accepting all combinations of training	62	33	11	32	91	29	0	33
% Unreported residency requirement	30	34	84	54	1	71	67	33
Ortho faculty supervision, %	2 (15)	3 (25)	9 (14)	4 (19)	30 (32)	0	0	0
Plastics faculty supervision, %	0 (0)	0 (0)	2 (3)	2 (9)	7 (7)	0	0	0
Ortho-plastic faculty supervision, %	4 (31)	3 (25)	6 (9)	5 (23)	48 (51)	0	0	0
Faculty specialty not apparent, %	7 (54)	6 (50)	47 (73)	11 (50)	9 (10)	7 (100)	3 (100)	3 (100)

*Note.* A-NZ = Australia/New Zealand; SA = South America; AME = Africa/Middle East; SD = standard deviation; ortho = orthopedics.

a Variable length programs had durations of 6 months with option to extend up to 1 year.

**Table 2.** Reported Program Supervision by Region and Trainee Type.

	No. of programs	Average number of supervisory faculty (SD)	Orthopedic faculty supervision only, %	Plastic faculty supervision only, %	Ortho-plastic faculty supervision, %
Global	218	7.4 (3.9)	50 (22.9)	11 (5)	68 (31.2)
USA	94	7.9 (3.7)	30 (32)	7 (7)	48 (51)
Europe	64	7.6 (5.3)	8 (13)	2 (3)	6 (9)
UK	22	5.9 (3.6)	4 (18)	2 (9)	5 (23)
A-NZ	13	4.8 (2.8)	2 (15)	0	4 (31)
Canada	12	6.0 (3.3)	3 (25)	0	3 (25)
Asia	7	—	2 (29)	0	1 (14)
SA	3	13 (-)	0	0	0
AME	3	6.5 (0.7)	1 (33)	0	1 (33)
Accepting ortho-trained residents	130	7.7 (3.7)	37 (29)	8 (6)	58 (45)
Accepting plastics-trained residents	120	7.9 (3.7)	28 (23)	8 (7)	58 (48)
Accepting general surgery-trained residents	53	8.3 (4.3)	18 (34)	18 (34)	23 (43)

*Note*. SD = standard deviation; ortho = orthopedics; A-NZ = Australia/New Zealand; SA = South America; AME = Africa/Middle East; n/a = insufficient data.

### Educational Format and Supervision

The relationship between fellow and faculty was specified in 44% of programs. Rotation through various practices was reported in 54% of these programs, and 15% reported a one-on-one mentorship/apprenticeship model.

Participation in didactic teaching modalities such as lectures, journal club, and other meetings were commonly specified expectations of Fellows in 62% and 50% of US and Canadian programs, respectively. These expectations were infrequently outlined in programs from AME (33%), SA (33%), Asia (14%), and Europe (3%), and absent in the United Kingdom and A-NZ. Teaching and supervision of residents/medical students was described in 67%, 49%, 33%, 15%, and 5% of programs in Canada, the United States, SA, A-NZ, the United Kingdom, respectively, but not in any program in Europe, Asia, or AME.

### Reported Program Goals, Objectives, and Clinical Competencies

**Figure 2.**
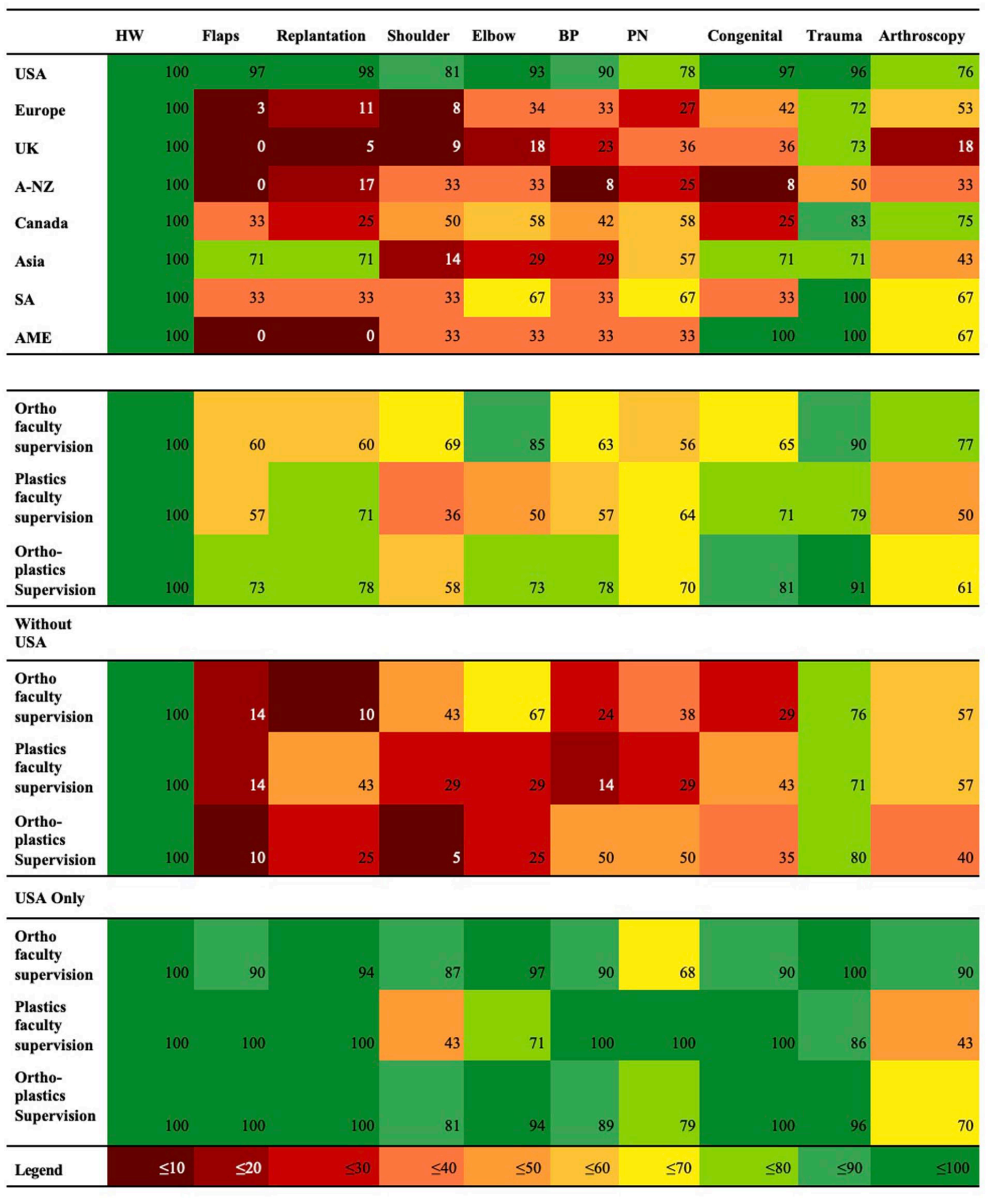
Heat mapping of program reported clinical competencies by region and Fellowship characteristics. *Note.* HW = hand/wrist; BP = brachial plexus; PN = peripheral nerve; A-NZ = Australia/New Zealand; SA = South America; AME = Africa/Middle East.

**Figure 3.**
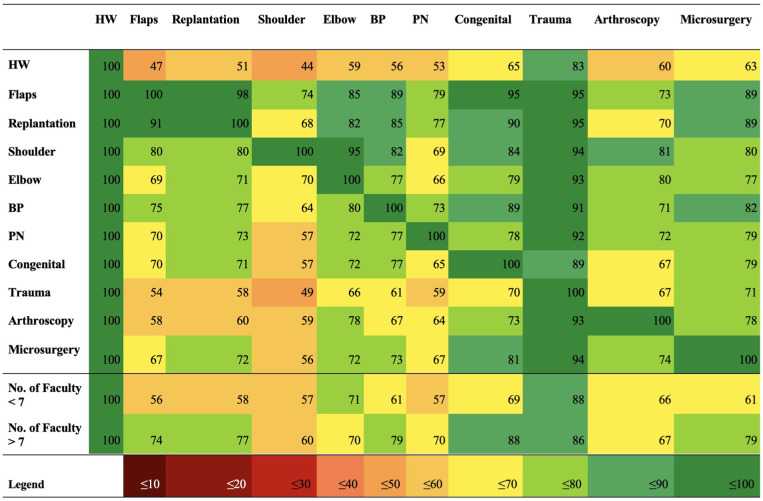
Heat mapping of correlated reported clinical competencies and faculty size. *Note.* Percentage values are provided for each time a hand surgery program website co-reports a clinical competency in the column with a clinical competency in the corresponding row. HW = hand/wrist; BP = brachial plexus; PN = peripheral nerve.

Of the 218 programs, 158 detailed general program objectives (Table 3). Descriptions often presented an emphasis on comprehensive training for management of upper extremity conditions, with 57 programs discussing this concept. Figures 2 and 3 display the reporting frequency of various competencies discussed by programs. We also focused on Microsurgery training, which was frequently described by Hand Surgery Fellowships (Table 4): common methods included completion of a microsurgical training course or access to microsurgical training labs.

**Table 3.** Program Goals and Clinical Competencies Reported Among Hand Fellowships.

	No. of programs with stated goals, %	No. of programs with specifically outlined clinical competencies, %	No. of programs with dedicated research time, %	No. of programs with research support, %
USA	74 (79)	35 (37)	24 (26)	51 (54)
Europe	40 (63)	1 (2)	1 (2)	0
UK	20 (91)	0	0	1 (5)
A-NZ	7 (54)	1 (8)	3 (23)	4 (31)
Canada	10 (83)	2 (17)	4 (33)	2 (17)
Asia	4 (57)	0	0	1 (14)
SA	0	0	0	0
AME	3 (100)	0	0	0
Accepting ortho-trained residents	99 (76)	37 (29)	30 (42)	55 (42)
Accepting plastics-trained residents	91 (76)	34 (28)	26 (41)	49 (41)
Accepting general-trained residents	36 (68)	16 (30)	13 (49)	26 (49)
Ortho faculty supervision	43 (86)	13 (26)	16 (38)	19 (38)
Plastics faculty supervision	10 (91)	1 (9)	1 (9)	1 (9)
Ortho-plastic Supervision	54 (79)	21 (31)	14 (44)	30 (44)

*Note.* A-NZ = Australia/New Zealand; SA = South America; AME = Africa/Middle East; ortho = orthopedics.

**Table 4.** Microsurgery Training Reported Among Hand Fellowships.

	No. microsurgery competency, %	No. microsurgery course, %	No. microsurgery lab, %	No. both course and lab, %	No. neither course nor lab and claiming competency, %
USA	84 (89)	38 (40)	39 (42)	21 (22)	7 (7)
Europe	31 (48)	1 (2)	0	0	30 (47)
Canada	7 (58)	3 (25)	0	0	4 (33)
A-NZ	4 (31)	0	0	0	4 (31)
Asia	4 (57)	1 (14)	1 (14)	0	2 (29)
UK	2 (9)	0	0	0	2 (9
AME	2 (67)	0	0	0	2 (67
SA	1 (33)	0	0	0	1 (33)
Ortho faculty supervision	33 (67)	11 (22)	17 (35)	6 (12)	5 (10)
Plastics faculty supervision	9 (82)	1 (9)	1 (9)	1 (9)	7 (64)
Ortho-plastic Supervision	49 (73)	22 (33)	18 (27)	11 (16)	9 (13)
Accepting ortho-trained residents	96 (76)	39 (31)	39 (31)	21 (17)	18 (14)
Accepting plastics-trained residents	92 (77)	37 (31)	38 (32)	20 (17)	17 (14)
Accepting general-trained residents	44 (83)	16 (30)	21 (40)	11 (21)	7 (13)

*Note.* A-NZ = Australia/New Zealand; SA = South America; AME = Africa/Middle East; ortho = orthopedics.

Thirty-nine (18%) programs reported a list of specific learning objectives, outlining individual clinical presentations and procedures for which Fellows were expected to gain proficiency. Common themes included establishing a detailed knowledge of anatomy and pathophysiology, fostering diagnostic and evaluation skills, and developing proficiency in surgical techniques.

Involvement in research was required in Canada (83%), the United States (71%), A-NZ (62%), the United Kingdom (55%), AME (33%), and Asia (29%), with 75%, 37%, 31%, 5%, and 0% specifically mentioning publication, respectively. Table 3 highlights 58 programs offering research support globally, comprising one or more of lab space (40), assistance research personnel (24), faculty mentorship (10), project funding (9), database access (6), specialized equipment (5), or office space (2).

## Discussion

The objective of this study was to characterize specific features, instructional methods, competencies obtained, and clinical objectives of hand surgery training programs on a global scale. Previous studies in hand surgery education have largely focused on data from the United States alone.^1,4,6,8,10,11,14,16-18^

### General Program Characteristics

The results of this study depict reports from 8 major geographical regions. Three of these regions—North America, Australasia, and Europe—are a focus of the article by Tonkin et al^19^ on trends in global hand surgery training. Our study found 1-year programs to be the most common, especially in North America. Six-month programs with or without the option to extend to 1 year were more frequently reported outside North America. This may be related to program structure, including the Fellows’ clinical role, number of learners, teaching approaches, and preferences and caseloads of the faculty supervisory team, which may influence time to proficiency. The United States demonstrated the highest uptake of general surgery residents, likely owing to overlapping residency training avenues in plastic and general surgery.^19,20^ Universally, programs are predominantly open to orthopedic-trained residents, and supervised by orthopedics-only or mixed faculty, as opposed to plastics-only. The balanced acceptance of both plastics-trained and orthopedics-trained residents in the United Kingdom reflects local practice of hand surgery as an “interface specialty.”^19^ The relatively lower acceptance rate of orthopedic-trained residents to plastics-trained residents in the United Kingdom may correspond to local notions of hand surgery as a mostly plastics-based specialty.

### Educational Format

There is a lack of literature addressing educational formats in Hand Fellowship programs. Common educational formats included rotation through various faculty practices, which was most prevalent in the United States. This may explain the ability of US programs to consistently offer exposure to different clinical competencies in hand surgery, as seen in Figure 2. Prominent involvement in resident/student teaching and didactic rounds within North America reflects the critical role of Fellows in medical education, and perspective on teaching as a vital skill and educational tool.

### Objective Programs Goals and Clinical Competencies

US programs most consistently stated program objectives and reported relevant competencies on their websites. This is likely the result of previously established requirements by the ACGME.^12^ Canadian programs frequently reported trauma and arthroscopy skills, while skills within the microsurgical domain were less commonly reported (Figure 2). This disparity may be due to the predominantly orthopedic nature of program supervision and relative nascency of hand surgery training in Canada. Programs beyond North America infrequently reported specific clinical competencies. Despite significant underreporting in these countries, trauma and arthroscopy were commonly described in the United Kingdom, Europe, A-NZ, AME, and SA (Figure 2).

Orthopedic-supervised programs more commonly reported training in elbow, shoulder, and arthroscopic surgery. Mixed or plastic surgery-supervised programs more commonly offered training in congenital, peripheral nerve, replantation surgery, and microsurgery. This may reflect the practice of faculty supervisors, based on surgical discipline. These skills are also congruent with the exposures of orthopedic and plastic-trained residents entering these programs, as outlined by the ACGME and case log studies.^8,9,21-23^ Competency in microsurgery was most infrequently reported in the United Kingdom. Given the low incidence in reporting overall, this may likely be the result of underreporting as opposed to general lack of competency in this area. Programs supervised by plastics-only faculty most commonly reported the microsurgical competency without mention of a lab or course—while underreporting may again be an issue; this may also be explained by the tendency of plastic surgeons to pursue microsurgical training, making microsurgical exposure more likely throughout the Fellowship itself, as opposed to a course or lab.

Figure 3 demonstrates the ubiquity of trauma cases in training programs. Shoulder was the least reported competency of training programs (45%), followed by flaps (47%), replantation (51%), peripheral nerve (53%), brachial plexus (57%), elbow, and arthroscopy (60%). Decreased reporting of shoulder, elbow, and brachial plexus surgery is expected, as many Hand Fellowships do not necessarily cover the proximal upper extremity, and programs that report these competencies commonly co-report the other (Figure 3). Notably, brachial plexus, elbow, and shoulder surgery are not US accreditation requirements, whereas arthroscopy and replantation are required by the ACGME.^12^ Similar reporting of these competencies necessitates further discussion on whether the proximal upper extremity should be considered in hand surgery accreditation guidelines.

Although flap and replantation surgery skills were less commonly reported overall, programs reporting them also reported most other competencies (Figure 3). Interestingly, programs with supervisory team size less than 7 faculty were less likely (~20%) to report flap surgery, replantation, congenital, brachial plexus, and microsurgery. This may be explained by the subspecialized care involved in microsurgical or severe trauma cases. These cases may be more likely performed at larger centers that are more equipped in all aspects of hand surgery. Notably, the average team size is skewed, and US programs consistently report above-average supervisory team size (Table 2).

### Relevance to Hand Surgery Training and Future Directions

Mapping Hand Fellowship characteristics on a global scale allows identification of common and therefore useful educational objectives and outcomes. This in turn validates the value of a Fellowship, with potential to inform employers and improve future hand surgery training models. The variability of reported obtainable clinical competencies across faculty specialization and geographical region highlights the need for Hand Fellowship programs to standardize their outcomes (and the reporting of these outcomes), which would enable surgeons to deliver a similar standard of care globally. In addition, the lack of available data for programs not recognized by a national regulatory body stands in stark contrast to formally recognized programs, further reinforcing the need for improved data reporting and standardization of Hand Fellowships, as well as transparent, updated online visibility.

In addition to offering insights into the standard requirements of Hand Fellowships globally, this work offers perspective on the future structure of Hand Surgery Training. The 2018 review by Tonkin et al^19^ of the history of hand surgery training in regions including the United States, the United Kingdom, Australia, and Singapore offers insightful debate on the past, present, and future structure of hand surgery training, namely from presidents of societies including the IFSSH, ASSH, British Society for Surgery of the Hand, and Singapore Society for Surgery of the Hand. While arguments have been made for co-existence of hand surgeons from various training backgrounds (ie, traditional training), others including Tonkin argue the possibility of an “expert in the upper extremity,” with integrated cross-disciplinary residency training in orthopedic, plastic, neuro-, and vascular surgery required to provide entirely comprehensive care—as is reported in regions such Singapore and Australia.^19^ The shift toward subspecialized care in regions such as the United Kingdom lies in the way of adoption of an “upper extremity surgeon” or hand-specific residency program, however.^19^

While findings from our study alone cannot substantiate movement toward or away from an integrated hand surgery residency model—or implementation of specific curriculum standards—consultation with current Fellows and correlation with our study data could provide insights into essential Hand Surgery Training components, which could guide the development of measurable outcomes, clinical competencies, and assessment of residency/Fellowship effectiveness in conferring expertise to the surgeon.^3^ Correspondingly, consultation with practicing hand surgeons would also help to contextualize this work and inform guidelines. Like the ASSH, the use of centralized application avenues more globally could facilitate the establishment of exchangeable Fellowship curricula.

### Limitations

A prominent limitation of this study regards the completeness of individual program descriptions globally. Descriptions often possessed variable amounts of detail. Program websites and postings may have been posted several years, or on rare occasions, decades prior, which may not represent the current state or objectives of these programs. The incompleteness and inaccessibility of US Fellowship websites was previously highlighted by Hinds et al^24^ and has been observed across geographical regions captured in this article. Moreover, while this study combined the claims between the ASSH descriptions and individual program websites, these could be compared in future studies.

This study inherently contains a North American bias. The use of browsing software from a Canadian address ultimately influences search results. The ASSH provides a comprehensive list of Fellowships and their key features, which is not consistent globally. Some regions do not routinely list affiliation with a regulatory body or Hand Society and programs were excluded as a result. Exclusion of non-English program descriptions also contributed to this bias, which was mitigated by use of translation tools. The small sample size of regions relative to the United States may limit the validity of study results.

The global scale of this study implies inevitable incompleteness of this dataset. It is likely that many fellowships evade the inclusion criteria specified in this study. New fellowships that may or may not be publicly shared on the Internet are continuously being developed across the globe, in different languages. Future studies to this effect would benefit from the use of AI assistive technologies to perform a uniform scan of available programs in different countries/languages and extract relevant data for more fulsome analysis.

## Supplemental Material

sj-docx-1-han-10.1177_15589447251406922 – Supplemental material for Global Hand Surgery Fellowship Education: Program-Reported Characteristics and Competencies Supplemental material, sj-docx-1-han-10.1177_15589447251406922 for Global Hand Surgery Fellowship Education: Program-Reported Characteristics and Competencies by Andrew D’Elia, Armaan Dhanoa and Barbara Jemec in HAND
