# Supplementary material for: Global Hand Surgery Fellowship Education: Program-Reported Characteristics and Competencies
Source: Hand (N Y). 2026 Jan 2:15589447251406922. Online ahead of print. doi: 10.1177/15589447251406922 (PMC12764421; doi:10.1177/15589447251406922)
Supplement: sj-docx-1-han-10.1177_15589447251406922 – Supplemental material for Global Hand Surgery Fellowship Education: Program-Reported Characteristics and Competencies [file sj-docx-1-han-10.1177_15589447251406922.docx]

**SUPPLEMENTAL MATERIALS**

**Table S1. Contents of IFSSH/FESSH Constituent Website**

|  | **IFSSH** | **FESSH** |  |
| --- | --- | --- | --- |
| Total No. Constituent Societies | 63 | 68 |  |
| No. Constituents with website (%) | 42 (66%) | 57 (83%) |  |
| No. working website links (%) | 36 (57%) | 44 (77%) |  |
| No. websites in english/translatable (%) | 16 (25%) | - |  |
| No. Websites with: |  |  |  |
| Info on clinical fellowships | 5 (8%) | 43 (63%) |  |
| Info on courses and/or continuing education | 26 (41%) | - |  |
| info on travelling/grant fellowships | 14 (22%) | - |  |
| info on journals/conferences/congresses | 29 (46%) | - |  |

**Table S2. Details of Excluded Fellowship Programs**

| **Region** | **Programs** | **Duplicates** | **Insufficient Data** | **Non-English/Translatable** | **Travel/Research Fellowships** | **No Affiliation** | **Closed/Expired** |
| --- | --- | --- | --- | --- | --- | --- | --- |
| UK | 7 | 2 | 1 | 0 | 2 | 0 | 2 |
| Europe | 19 | 1 | 3 | 1 | 1 | 13 | 0 |
| Asia | 20 | 0 | 0 | 1 | 7 | 12 | 0 |
| Australia | 3 | 2 | 0 | 0 | 0 | 1 | 0 |
| Africa | 2 | 0 | 2 | 0 | 0 | 0 | 0 |
| Middle East | 4 | 0 | 0 | 0 | 0 | 3 | 1 |
| South America | 8 | 0 | 0 | 0 | 0 | 8 | 0 |
| Total | 63 | 5 | 6 | 2 | 10 | 37 | 3 |
